# Use of the adult attachment projective picture system in psychodynamic psychotherapy with a severely traumatized patient

**DOI:** 10.3389/fpsyg.2014.00865

**Published:** 2014-08-05

**Authors:** Carol George, Anna Buchheim

**Affiliations:** ^1^Department of Psychology, Mills CollegeOakland, CA, USA; ^2^Institute of Psychology, University of InnsbruckInnsbruck, Austria

**Keywords:** Adult Attachment Projective Picture System, psychoanalysis, psychotherapy, trauma, adult attachment

## Abstract

The following case study is presented to facilitate an understanding of how the attachment information evident from Adult Attachment Projective Picture System (AAP) assessment can be integrated into a psychodynamic perspective in making therapeutic recommendations that integrate an attachment perspective. The Adult Attachment Projective Picture System (AAP) is a valid representational measure of internal representations of attachment based on the analysis of a set of free response picture stimuli designed to systematically activate the attachment system (George and West, 2012). The AAP provides a fruitful diagnostic tool for psychodynamic-oriented clinicians to identify attachment-based deficits and resources for an individual patient in therapy. This paper considers the use of the AAP with a traumatized patient in an inpatient setting and uses a case study to illustrate the components of the AAP that are particularly relevant to a psychodynamic conceptualization. The paper discusses also attachment-based recommendations for intervention.

## INTRODUCTION

### PRESENTING SYMPTOMS

Gloria, a mid-aged patient, appeared restless and somewhat distrustful initially during her diagnostic clinical interview; she gained trust during the interview because the therapist’s open and direct approach seemed to defuse the patient’s fear of a discussing trauma therapy. She reported during this interview that 5 years ago she had a gruesome experience with a therapist who had suggested immediately at their first meeting that she begin trauma therapy for her rape experience. Gloria was terrified and she quit therapy.

When the second author first saw Gloria for the attachment assessment, her symptoms indicated a strong dissociative avoidance disorder, which also included headaches, fainting in stress situations, and memory loss. She had been in a car accident about 1 year prior to this time and, as a result, she felt for the first time in her life that treatment would be appropriate and she contacted a psychosomatic hospital. Gloria was diagnosed with post-traumatic stress disorder (PTSD; DSM-IV) with high dissociative states (amnesia) and a pain disorder.

### HISTORY OF RELATIONSHIP TRAUMA AND COURSE OF ILLNESS

Gloria lived in an intact family with her parents and three younger siblings until her parents divorced at age 5, but she provided no details about her childhood before this time and would not speak at all about her biological father. Gloria and her siblings lived predominantly with their mother after the divorce. Her mother remarried 5 years later when Gloria was 10 years old and she viewed her stepfather as her “actual” father. She described him as being humorous, loved, and trusted, but she also described him impulsive, irascible, and argumentative. Gloria seemed insecure about her stepfather’s acceptance, wondering how far she could push him before he would break. Would he accept her even if she acted like a wild child? Gloria stated that “once a week I pushed him until he burst,” and she told how she tested him with “mischievousness” so as to push her stepfather into beating her. Gloria’s deliberate misbehavior and her stepfather’s beatings were central to their relationship.

Gloria’s first major traumatic experience as a sadistic rape in late adolescence. The only details that she provided about her rape was that it occurred during daytime and that she did not know the rapist. After the rape, she began around 3 weeks later to have sudden headache and fainting attacks, fainting as much as three times a day. She also developed chronic dissociation experiences. Gloria’s symptoms appeared to be associated with feeling of being exposed and to school or performance-related pressure. Although these problems persisted, she did not seek psychological treatment. Her symptoms, especially fainting, diminished when she studied abroad. Her symptoms reoccurred after returning home 2 years later, however, and she decided to go back abroad.

Gloria’s second major traumatic experience was at 30 years old when her boyfriend of 2 years died in an accident. Gloria had separated from him shortly before his death, the reason being that she was no longer able to tolerate physical closeness. She felt severely guilty about his death and her guilt had masochistic qualities. As a result, she did not have another intimate relationship for many years.

Gloria had recently experienced a third trauma prior to her decision to seek treatment. She had been in a serious accident in which she had been thrown out of her car and into the air rendering her unconscious. She was thought at first to be dead. Her physical injuries included three spinal discs and a strain on the cervical spine and her fainting episodes increased to many episodes a day. Although Gloria reported in her initial interview, almost proudly, saying “I survived this,” and she had since was unable to work.

Gloria felt that her symptoms had become debilitating, and she noticed that her fainting spells seemed related to stress. Her headaches had become so severe that she risked becoming unconscious. She was not able to recall what preceded the headaches and she could not remember any indicators associated with their onset, such as less debilitating headaches or other physical warning signs. Gloria described herself as being on *autopilot*. This “defensive mechanism” had saved her life more than 20 years ago, but now this automatic mechanism was out of her control.

Gloria had not allowed herself to think about this until she entered treatment and her treatment goal was “to get rid of it.” She had a stiff commitment to being strong and carrying on. “I want to function. I will get through this. I want to be able to work. I have worked for many years to wipe out the traumatic event, to get rid of it, to repress it.” This perspective had dominated her life and kept her moving forward. She was frightened of not being able to be in control of her symptoms and the prospect of becoming dependent on the pain medication prescribed to combat her severe headaches.

## BACKGROUND

### ATTACHMENT AND PSYCHOANALYSIS

Bowlby (1969) was a prominent psychoanalyst to use ethological concepts to describe the infant’s biologically predisposed attachment to a primary caregiver. He viewed relatedness in early childhood as a primary and independent developmental goal that is not subservient to a physiological needs (e.g., hunger) or psychoanalytically defined primary processes. The infant is perceived from an interactional perspective, with a focus on the relationships with primary attachment figures. Attachment theory maintained some foundations of psychoanalytic theory (e.g., the developmental point of view) and there is a strong literature that discusses the divergences and convergences of psychoanalysis and attachment theory (e.g., Diamond and Blatt, 1999; Fonagy, 1999, 2001; Slade, 2000; Gullestad, 2001; Steele and Steele, 2008; Eagle, 2013) and also developed some aspects further, particularly the delineation of the internal world (Diamond and Blatt, 1999). Fonagy’s (1999, 2001) overview of the intersection of these two approaches demonstrated that the relationship between attachment theory and psychoanalysis is more complex than adherents of either community have generally recognized. This paper addresses some of these complexities by integrating attachment assessment using the Adult Attachment Projective Picture System (AAP) in psychodynamic psychotherapy in an adult traumatized patient.

George and Solomon (1999) proposed that one major difference between psychoanalysis and attachment theory falls in the description of forms of defensive processes. Traditional psychoanalytic models provide a complex constellation of defenses to interpret a broad range of intrapsychic phenomenon, including phantasy, dream, wish, and impulse (e.g., Horowitz, 1988; Kernberg, 1994). Attachment theory delineates two basic processes that manifest in three forms. According to George and Solomon (1999, 2008), Bowlby defined defense as forms exclusion directed to modulating difficult and anxious experiences with attachment figures, and the child’s experiences with incomplete or failed bids for parental protection, care, and comfort. He defined defenses in terms of two qualitatively distinct processes: deactivation (retaining elements of intellectualization and denial) and cognitive disconnection (retaining elements of splitting). George and Solomon (1999, 2008) pointed out that under normal circumstances these two exclusion processes are associated with goals to maintain physical and psychological proximity in the attachment-caregiving relationship under conditions when the child’s experiences with the attachment figure are less than satisfying. George and Solomon refined Bowlby (1980) model suggesting that deactivation and cognitive disconnection organized and supported at least minimal forms of representational, behavioral, and emotional regulation. Bowlby’s (1980) proposed that these forms defensive exclusion functioned to segregate (akin to repression) memory, affect, and experience when the attachment figure was not available, conceiving of an extreme process he termed “segregated systems.” Segregated systems were thought as associated with the painful and chronic distress experiences, such as those that accompany loss. Bowlby posited that segregated systems were the intrapsychic root of symptoms related pathological mourning and severe psychopathology. Attachment theorists have since demonstrated that segregated systems are associated with experiences of failed protection, attachment trauma, and disorganized/dysregulated attachment behavior and representation (Solomon and George, 2000, 2011; George and West, 2012).

Consistent with a psychoanalytic approach, some attachment theorists have suggested that utilization of defensive process models is needed to provide a complete picture of the emotional and behavioral regulation processes individuals develop from their childhood relationships with attachment figures (Cassidy and Kobak, 1988; George and Solomon, 1999; George and West, 2012). Further, George and West (1999) concluded, “In order to understand the relationship between adult attachment and mental health risk we need to examine the attachment concepts of defense and segregated systems, the mental processes that define disorganization” (p. 295). These theorists operationally defined Bowlby (1980) basic defense scheme as a central element for evaluating representational patterns of attachment using the Adult Attachment Projective Picture System (George et al., 1997, 1999; George and West, 2012). Suggesting that these representational structures have developed under conditions of attachment trauma (abuse, loss, failed protection), the concept of segregated systems is fruitful to explain some forms of relationship-based psychopathology in adults (George and West, 2012).

The discussion that follows provides some ideas about using attachment concepts in clinical work by showing how the perspectives of a psychoanalyst and attachment assessment may improve the understanding of an individual case of a traumatized patient with the diagnosis of a PTSD with dissociative states (e.g., fainting in response to stressful situations).

### POSTTRAUMATIC STRESS DISORDER

The lifetime prevalence of PTSD in Germany has been found to be 1.3% with a female-to-male ratio of 3.25–1. Traumatized patients are frequently misdiagnosed and mistreated in the mental health system. The number and complexity of the symptoms lead to fragmented and incomplete treatment. PTSD patients are vulnerable to become re-victimized by caregivers because of their difficulties with close relationships. Severely traumatized PTSD patients (complex trauma) develop difficulties in modulating arousal and show signs of severe affect dysregulation (e.g., aggression against self and other, and problems with social attachment and dissociative states).

Dissociation, defined as a deficit of the integrative functions of memory, consciousness and identity, is often related to traumatic experiences and traumatic memories (Liotti, 2004; Kihlstrom, 2005). During clinical interviews, dissociation is suggested either by such a degree of unwitting absorption in mental states that ordinary attention to the outside environment is seriously hampered. Dissociation can be accompanied by a sudden lack of continuity in discourse, thought or behavior of which the person is unaware (supposedly due to intrusion of dissociated mental contents in the flow of consciousness). Thus, for instance, a dissociative patient may suddenly interrupt her speech during a therapeutic session, stare into the void for minutes, and become unresponsive to the therapist’s queries as to what is happening to her. Or a patient suffering from PTSD may suddenly utter fragmented and incoherent comments on intrusive mental images (usually related to traumatic memories) that surface in consciousness and hamper the continuity of the preceding dialog with the therapist. In the most extreme variety of dissociation (Dissociative Identity Disorder), an alternate ego state may appear during the clinical dialog, reporting (sometimes with an unusual tone of voice, e.g., like a child) memories of childhood abuse of which the patient has previously been totally unaware, or expressing attitudes and beliefs quite extraneous to the patients’ personality (Liotti, 2004).

Furthermore, shattered meaning propositions predominate. Trust, hope and sense of agency is accompanied by social avoidance, with loss of meaningful attachment and therefore lack of participation in preparing for future (van der Kolk et al., 1996).

### DISORGANIZED-DYSREGULATED ATTACHMENT AND DISSOCIATIVE SYMPTOMS

The founding premise of attachment theory is that stress, especially traumatic stress, produces a strong desire for proximity to and comfort by attachment figures; this desire is built into human biology as a survival safety mechanism and the mechanism is functions unchanged throughout the life span (Bowlby, 1969). Attachment experience shapes the ways in which individuals manage stress and are especially important when individuals experience a traumatic event (Bowlby, 1973, 1980; Schore, 2001). When attachment is secure, individuals know how and when to seek attachment figures and develop internal representations of self as deserving of care. Attachment security fosters confidence and trust that figures are available, empathic, and sensitive to their needs; security is a buffer or resilience factor that supports recovery from trauma (Bowlby, 1980; Schore, 2001). When attachment is insecure, emotional and behavioral reactions when distressed may be made even more painful by unconscious evaluations that wishes for comfort are illegitimate. Insecurity may result in additional painful interactions with the attachment figures rather than the functional comfort and protection for which attachment was intended (Bowlby, 1980). Insecurity fosters anxiety, anger, and fear, and increases the risk of developing trauma-related *emotional* disorders (Bowlby, 1980; Adam et al., 1995; Dozier et al., 2008; George and West, 2012). Extreme forms of insecurity are associated with the breakdown of attachment and caregiving regulatory mechanisms risk emotional and homeostatic dysregulation, often termed disorganized attachment (Solomon and George, 2011). The risk of dysregulation is heightened when attachment relationships are threatened or threatening, such as parental loss or psychiatric debilitation or maltreatment (Lyons-Ruth and Jacobvitz, 2008). George and West (2012) defined events such as these as attachment traumas, events that involve terrifying threats to the integrity of self or attachment relationships. Attachment disorganization, conceived in terms of mechanisms of dysregulation and attachment trauma, has been shown to predict vulnerability to severe psychiatric symptomology, including dissociative symptoms (Lyons-Ruth and Jacobvitz, 2008; Weinfield et al., 2008; Solomon and George, 2011).

Liotti (2004) found the metaphor of a “drama triangle” useful in thinking about the intersection between dissociation and disorganized attachment. The dissociation triangle addresses how disorganized attachment fosters dissociative mechanisms that create incompatible and separate representations of self as victim, rescuer, and persecutor. The child’s representation of the attachment figure is represented in a conflicting manifold way. On the one side, the attachment figure is represented as source of the child’s fear, the self as a victim of attachment figure as persecutor. On the other side, the attachment figure by virtue of being the child’s biological protector is viewed as the child’s source of safety and protection (rescuer). In the child’s mind, representation of self and attachment figure shift among these three incompatible models that are too complex to be synthesized into an integrated model of self. Liotti’s model provides us with an integrated psychodynamic and attachment approach to our first questions concerning Gloria’s illness, questions regarding the childhood origins of her episodes of near unconsciousness and her inability to ask for help following traumatic assault.

Fearon and Mansell (2001) examined cognitive perspectives on unresolved attachment in patients diagnosed with PTSD. They proposed that unresolved loss, as defined in attachment assessment during interview, involves intrusion avoidance phenomena similar to those of PTSD. Specifically, they develop a model based on unresolved loss that involves the failure to integrate representations of self and the world following a loss. The features of unresolved loss can be understood as emerging as a result of the activation of unintegrated representations of the loss experience and cognitive and behavioral avoidance processes. In this model, the sudden intrusion of memories, cognitions, and emotions associated with the loss automatically captures attention and initiates behavioral dispositions that are incompatible. With regard to attachment, the authors suggested that this was the mechanism that interfered with caregiving behavior. Lack of attentional resources and incompatible response tendencies can also result from safety behaviors directed at avoiding the perceived negative consequences of activating trauma memory. The authors proposed that these processes offer a novel way of understanding the disturbances in behavior and speech that are evident in mothers who are designated as unresolved with respect to loss.

This suggests that representational attachment measures, like the AAP, can provide a good understanding of the movement that the client might be making toward empowerment, integration, or understanding. Thus, even if a patient’s overall attachment is unresolved (i.e., dysregulated), there may be indications in their responses to the AAP stimuli that suggest they are moving toward mental organization. Given the negative outcomes that are associated with abuse, focusing on resources and defensive strategies is arguably important for therapeutic recommendations.

## DISCUSSION

Looking at this case from both a psychodynamic and attachment point of view, we ask a complex set of questions seeking to understand how childhood attachment experience and trauma were related to the patient’s symptoms and her refusal to seek treatment. Were Gloria’s experiences with childhood attachment figures traumatic and how might early experience block her from seeking help? Was Gloria’s chronic denial of emotional pain related to her rape? Why were somatic symptoms – severe headaches and fainting – her only way to express pain? And finally, why had Gloria retreated with her ailments and refused to address her trauma?

The discrepancy in Gloria’s descriptions is noteworthy. She idealized her stepfather by saying how much she loved him, while simultaneously describing his repetitive harsh spankings. From a psychodynamic viewpoint, these two object-representations are split and unintegrated. We speculated, therefore, that her stepfather’s beatings were the answer she was looking for to confirm her experience as being recognized and loved.

The attachment perspective contributes added depth to this observation. The juxtaposition of love with dysregulating painful parental rage are the foundation of segregated systems (i.e., repressed and dissociated experience), defined as unconscious representational processes that become obstacles to grieving trauma and foster psychosomatic symptoms (Bowlby, 1980; George and West, 2012). According to attachment theory, attachment figure proximity, even proximity involving pain, is a better attachment solution than feeling isolated or abandoned (Bowlby, 1973; George and West, 2012). This thinking then permits us to better understand Gloria’s representation of self in terms of the dissociation triangle described earlier. The quality of this daughter-stepfather relationship would be described as punitive-controlling attachment, a dysregulated form of attachment in which children seek to conquer feelings of abandonment and helplessness through parent-child combat (George and Solomon, 2008). The child in a punitive attachment relationship reciprocally plays out the roles of persecutor and victim.

Gloria’s description of her mother is limited. She described her mother as distant and busy with her family and job. Her mother would “let things go” but also punished her. Unlike her stepfather’s beatings, her mother used the “silent treatment,” interpreted as withdrawal of maternal love and engagement. Gloria recalled, “She simply did not talk with me for many days.” Once again, attachment theory provides additional depth to Gloria’s experience with her mother. This form of parental withdrawal has been shown to be a reaction to parental feelings of being out of control and helpless; the child’s experience is for the parent to inexplicably become psychologically invisible and vulnerable (George and Solomon, 2008). Faced with psychological abandonment, the silent treatment fosters a relationship in which the child must be very careful not to instigate the parent’s withdrawal. Gloria would know quickly that her misbehavior-enraged script with her father was a dangerous script with her mother. Children in these situations develop precocious sensitivity and caregiving skills toward the parent; feelings of helpless abandonment are regulated by role reversal (George and Solomon, 2008). The child becomes a skilled caregiver, hypervigilant and seeking to rescue the parent from her pain. Gloria likely assumed the role of rescuer with her mother, completing the disorganized attachment-dissociation triangle.

In describing her life story, Gloria remembers how she enjoyed her parents’ busy work schedule. Busy with their own life, she told how it provided her with a sense of freedom and independence. “If they are not caring, then at least I can do what I want.” Her independence often fostered dangerous or senseless recklessness. Rebelling against and refusing to accept her parents’ rules, she described herself as wild and out of control. She did what she wanted, including dangerous things that she now thought were stupid (e.g., climbing up the chimney, jumping on to the train tracks). When her parents sent to her to her room and forbade her to go out, she had climbed out of the window. She endured her punishment bravely and punishment did not deter her from doing these things again. According to attachment theory, Gloria’s dangerous and defiant behavior in adolescence is viewed as attachment behavior (Allen, 2008); and indeed, even though her parents’ response was punishment, Gloria achieved the connection to attachment figures she craved.

In her quest to be strong and rid herself of her symptoms, Gloria did not understand that her fainting was a defense mechanism. Fainting means survival and it was very likely that her trauma and fainting attacks were intertwined. The intersection of psychoanalysis and attachment theory is survival. From an attachment perspective, confronting her pain would threaten her fundamental ability to survive. From a psychoanalytic perspective, confronting these symptoms could mean her inner death and threatened survival may explain her resistance to the former therapist who had suggested trauma therapy.

### ADULT ATTACHMENT PROJECTIVE PICTURE SYSTEM: GLORIA’S ADULT REPRESENTATION OF ATTACHMENT

The AAP was administered after Gloria had been in inpatient treatment for 2 weeks. Her adult attachment classification was judged to be pathological mourning for unresolved trauma. Pathological mourning is a state of chronic mourning that endures for years because trauma produced segregated systems block completion of the mourning, Mourning in attachment theory is defined by conscious awareness and re-organization of memories and feelings related to trauma, which leads to a representation of self that integrates current reality with the past (Bowlby, 1980; George and West, 2012). Gloria’s AAP responses demonstrate the prominent role of attachment trauma in her representation of self and attachment relationships. The unresolved designation associated with her classification group signifies that she is not able to maintain regulatory processes to manage pain and fear. In Gloria’s case, we see her attempt to keep attachment trauma walled off or segregated as fortified by defensive deactivation. When working well, this form of defensive exclusion neutralizes and shift attention away from distress and pain (George and West, 2012). The wall breaks though and Gloria succumbs to dysregulation.

The AAP assesses attachment by asking individuals to respond to two kinds of attachment situations. One situation portrays individuals alone. The alone stimuli provide evidence of the capacity to develop strategies to cope with stress in the absence of any visual cues for potential solutions, including no visible portrayal of potential attachment figures (George and West, 2012). Alone responses are evaluated for potential agency of self (capacity for internal integration or constructive action) and connectedness to others, especially attachment figures. The other situation portrays individuals in potential attachment-caregiving dyads. These scenes are evaluated for synchrony, evidence relationship of reciprocity, sensitivity, and mutuality. We first discuss Gloria’s representation of self in response to alone AAP stimuli and then describe representations associated with the AAP dyadic stimuli.

It was Gloria’s representation of the alone self in *Bench*, the fourth AAP picture stimulus, in which she becomes dysregulated and attachment representation is “unresolved.” The stimulus figure is typically interpreted as an adolescent, drawn with legs and barefoot feet pulled up off the ground and arms loosely wrapped around the legs. The isolated vulnerability of this figure dysregulated her beyond repair. She told the following story, trauma designated in italics:

“Uh, a young woman, she is sitting on a bench and is very sad and unhappy. She is *desperate* and doesn’t know how to help herself. *She sees no way out and feels that no one helps her*. Feels left alone. *Helpless*, *stranded* and lonely. I don’t know. I have no idea. But I don’t know if it ends well, she is very sad. She may have the strength to pull herself out somehow. But if she has bad luck, then not. And then she will, *she will suffer further*.”

The young woman (projected self) is desperate, helpless, and suffering. She is confused and cannot envision life without suffering. The references to desperation, helplessness, and suffering are AAP indicators of severe traumatic content and emotional dysregulation. Gloria demonstrates no clear agency of self, unable to describe change or moving forward. Her representational self is void of a sense of connection to others. She succumbs to despair.

One common deactivation strategy in the AAP is evidenced by descriptions of sleep. Representational (and real) sleep is effective in that events can neither be detected nor processed. Sleep represents a deactivating defensive posture that filters out the details of distressing experiences from consciousness. Deactivation is central to maintaining segregated systems (Bowlby, 1980). We see how Gloria segregates images of stress-related dissociation (italics) with deactivation (underlined) in her *Window* response. *Window*, the first stimulus in the AAP set, portrays a rear view of a girl looking out a picture window.

“It is night time and the child is awakened, that is why she doesn’t have shoes on and is standing at the window and looking out. She is sad. Yes, she wishes I think that there actually was someone there with her but there is no one there now and that’s why she looks outside, so as if to say, yes, there is certainly someone outside that cares for me. She feels lonely and perhaps looks toward *the stars because they are comforting. But there are no stars above, or at least one can simply not recognize them*. At some point the child goes away and goes back to sleep, but I think when she grows up then she will go somewhere out in the world in order to find something.”

The most common responses to this scene are non-threatening descriptions of a child awakening in the morning to go outside to play or go to school (George and West, 2012). By contrast, Gloria’s response is a representation of self as desperately alone and without comfort. The child wishes for somebody, anybody, to care for her. Attachment theory views the girl’s “yearning and searching” as a natural response to separation and being alone; it is also associated with the initial phase of mourning loss of an attachment figure (Bowlby, 1980). In the absence of agency and connectedness, Gloria describes the girl’s solution with the haunting image of looking for comfort in galaxy beyond. This image is surreal and is evaluated in the AAP as a form of derealization. George (2013) found such AAP images were found in the AAP responses of individuals with severe childhood trauma. Gloria copes by returning to a deactivated mental state. By going *back* to sleep, the girl re-orients her attention away from just enough to create the image of being able to move forward in life. Although it contains Gloria’s dysregulated mind state, this image is fragile because Gloria fails to describe any concrete coping actions.

The last stimulus in the AAP set is an alone scene that portrays a child in the corner, drawn with solid lines that designate perceptual boundaries that potentially confine the child. The child is turned askew into the corner with one arm reaching outward away from the corner. Gloria told a story of trauma and self-protection, with trauma (in italics), personal agency (underlined), and her interpretation (in brackets) designated in her response:

“A small child stands in a corner and cannot get away. He defends, fends off with his hands. And he looks to the side and down because he tries in this way to protect his face or also not see what is coming next…. he only thinks, hopefully it will not be so bad. Afterward? He will be *beaten*. [It doesn’t help him that he fends against it… he should not have tried to avoid it because then he will be even more severely punished.] Yes, the scene repeats itself again and again. Until the child is someday simply big enough and can run away. *And he will surely never do that because he knows what it feels like to stands in the corner and not be able to return and get away*. So, he will have to get over it.”

Gloria’s final alone response describes the helplessness of abuse, the defining quality of her relationship with her stepfather. She describes a child who is helplessly trapped in the cycle of abuse. Important in her response, however, is the description of self has having the personal agency of protection. In those moments of abuse, this child was able to protect himself, a capacity to act. This was likely the source of Gloria’s capacity to go forward in life. What we see too though is that she currently sees the uselessness of the trying to protect one’s self. This may have been the impetus for seeking therapy. Unable to escape, she has not been able to “get over it.” The child’s agency in *Corner* did not change anything and Gloria’s overall representation of self in the alone stories demonstrates that she caught in a cycle of pathological chronic mourning for attachment trauma.

Gloria’s dyadic attachment representations demonstrated some sense of self as finding functional care from attachment figures. Her response to *Bed*, however, describes how the absence of functional care that leads to relationship failure. The *Bed* scene portrays a child sitting up in bed reaching toward the opposite end of the bed where the mother is seated. Gloria’s *Bed* story describes the sequelae of consistent maternal rejection. She told the following story in response to *Bed*; rejection is indicated by underline:

“That is clearly a night situation. The child has been sent to bed. The mother sits with him and the child wants very much like to be embraced, but the mother can probably not do that. She doesn’t make any sort of preparation to take the child into her arms. She is probably not in the position to do so. She loves her child but she can simply not be so, so loving or express her love so. With physical contact or something like that. And the child does not yet understand that and would very much like to be embraced. And over time he will feel cast off and will react similarly himself. The child simply thinks that he would like to be held by the mother. The mother thinks, I don’t want to do that. Or I can’t do that, it isn’t necessary. You have everything that you need. The child, so the relationship between the mother and the child will be in time one where the child doesn’t ask for it anymore. But I remain an optimist. The child will at some time find someone that can offer this loving and can also express it. And then he will be fine. But that all still takes time.”

This response evidences the process of how rejecting a child’s attachment bids creates distance in the relationships and extinguishes the child’s capacity to express its attachment needs. Bedtime signals separation for a child and naturally activates the need for comfort or at least a functional connection with the attachment figure (Bowlby, 1973). Gloria describes her need very clearly. She also “sees” how the mother’s withdrawal fosters rejection over time and extinguishes the child’s ability to ask. The need remains, as indicated by the suggesting that the boy will find a loving person who can provide what he needs. Feelings of intimacy and real connection, however, are sacrificed as it is only through mutual enjoyment and sensitivity that a child comes to know real intimacy (Bowlby, 1969). The relationship distance that results from rejection helps deactivate the distress of failed intimacy. It is like this experience that contributed to Gloria’s decision to leave her boyfriend.

Gloria’s overall AAP response patterns demonstrated a representation of self as helpless, desperate, abandoned, managed by deactivating defenses that created representational and relationship distance to shift her attention away from her pain. It is notable that none of these stories mirrored the independent or rebellious self she described during her clinical interview. Her AAP responses get beyond her desperate attempts to “get rid of” her feelings and demonstrate how frightened and helpless she really is. Gloria is caught in what seemed to be an endless cycle of trauma. Without protective and caring attachment figures, representational distance (deactivation), blurred by a positive smoke screen (hope to grow out of her circumstances), her only regulating mechanism is self-protection, “fending off” distress. We can see how dysregulation and chronic mourning have played out in her life. Adolescent and adult trauma combined with the threat of feared addiction to medication seemed to have rendered her helpless, likely because they were out of her control and she was helpless to draw on the control strategies she had developed during childhood with her parents. On a positive note, Gloria’s hope that she will someday find a caring relationship may finally support a commitment to developing a therapeutic relationship.

### THE PSYCHODYNAMIC VIEW

Attachment and psychodynamic perspectives are mutually informing. From a psychodynamic perspective, Gloria had learned early on to repress negative feelings, not to be noticed, to be “independent,” and to be outwardly “in control” when she was frightened. This coincided with her primary response mode – to endure and to pass over painful physical or emotional signs.

After her rape, a friend convinced Gloria to go to the police and to make a statement against the perpetrator. Ashamed and feeling that the police officer was insensitive to her situation, Gloria said that she was attacked but was able to get away, but she later retracted that statement. This retraction coincided with the onset of her severe headaches and the dissociative fainting episodes. She denied the severity of the trauma by not talking about it to anybody and suffered the consequences.

Freud (1920) defined “trauma” (Jenseits des Lustprinzips) as an overwhelming stimulus experience in response to which a healthy psyche cannot defend itself. The feeling of total helplessness compared with the traumatic experience sets the point of crystallization for further trauma. Helplessness is a difficult feeling to endure. This is presumably the reason why the psyche is not able to easily become peaceful after an overwhelming occurrence. Instead it tries on the one hand to hinder the return of the memory of the trauma in order to protect itself from further traumatization. On the other hand, there is a type of pressure to often deal with the incident in order to be able to find the fault of this threatening experience at least in thought, and perhaps in that to reach a process whereby the helplessness of the experience could be conquered through a new security or certitude. The traumatized person rocks back and forth between two opposite conditions – the “renunciation” (avoidance, dissociation) of the occurrence and the pressure of having to constantly remember it (intrusion). From the psychoanalytic viewpoint, these two opposite motivations deal in the classical sense with an intra-psychic conflict. Modern psychodynamic theory is derived from this trauma model.

The model emphasizes the effects of early development, especially physical and sexual trauma, with the simultaneous lack of protection of the child on psychic development. Up to now, research has shown that early traumatization is associated with deficits in the ability to steer intense affects highly unsettled by their attachment abilities (analogous to dysregulation risk associated with attachment disorganization). The effects of early traumatization often include attention and concentration deficits, antisocial behavior, the presence of physical symptoms as substitute expressions of emotional problems (somatization), as well as deep seeded hopelessness and a lack of basic trust. Early trauma has the most debilitating the consequences because, traumatic experience threatens the stability and the differentiation of the personality structure. The development of its affects, self confidence and trust requires a constant environment that supports stable and positive relationships to the parents.

This perspective suggests that it is reasonable to believe that the origin of Gloria’s vulnerability was her repeated beating by her stepfather combined with maternal psychological absence. She had learned at an early age to internalize this experience and interpret beating as the deserved and expected consequence of mischief. She could neither acknowledge nor speak about trauma under these conditions. Instead, she blamed herself and rendered these experiences taboo for a long time.

### TREATMENT SUGGESTIONS

Gloria’s response to starting trauma therapy was traumatic in itself. She did not want to look inside and remember the rape that she has tried to repress for more than 20 years. Since the fainting attacks are probably strongly connected to the trauma, an avoidance of treatment of the trauma would probably not improve her disease. A premature treatment of the trauma without an established working alliance would however be contraindicated, because of Gloria’s “gruesome” previous experience.

We would suggest the following key tasks in a psychodynamic treatment plan for this patient based on the AAP:

(1) Establish a secure base for this patient. This would mean not being intrusive and postpone delving into the rape experience until an alliance was established. Gloria’s unresolved-pathological chronic mourning classification on the AAP is also supports delaying exploring trauma and parenting failures because they are disorganizing and she is not able to contain feeling desperate, stranded, and helpless. She could not trust others to help her, and this would extend to an inability to trust her therapist. Rather, building on the hope Gloria alluded to in the *Bed* response bodes well for beginning the therapeutic relationship to build an alliance.

Part of the secure base therapist position would include helping Gloria develop a more integrated self-object representation. Gloria’s chronic representation of self as embodied the classic contradictory themes, as evidenced on the AAP. Her representation of self juxtaposes self-protective capacity with helplessness. One view was the self as frightened and helpless: I am frightened and helpless and cannot tolerate or verbalize it. The corollary theme was the self as having the capacity for protection: “I can protect myself and survive.” What becomes clear is that deactivation defenses neutralize and turn her attention away from her pain, thus maintaining trauma segregation and prevent her from understanding why she becomes dysregulated, “I dissociate and don’t know why.”

Other feature of the secure base position would help Gloria change her representation of object; that is, change the idealization of the stepfather to become more realistic. She would also need to accept the fact that, in attachment theory terms, her mother rejected her and did not protect her enough, which is why she had to learn to protect herself. Her avoidance, facilitated by deactivating defenses, is a reasonable response to her experience but is a maladaptive.

(2) Diminish deactivating defenses. Strengthen Gloria’s ability to tolerate negative feelings. This can help her face and accept the physical signs of distress in order to reduce the autopilot autonomic reactions. This can also help mitigate feelings of blame and foster increased ability for affect tolerance.

(3) Re-organize attachment dysregulation that is strengthened by deactivation. Help Gloria not reject and be frightened intimacy and closeness, and accept relationships with an authentic self who has weaknesses.

The therapist should be careful of being silent and appearing anonymous, since, for Gloria who is already neutralized and deactivated, this behavior would mean rejection. With this patient, it would be expected that the silence of the analyst could activate the previous mode with the mother, as shown in the Bed story and described in her childhood as her mother’s withdrawal of love: “She did not speak to me for days.” One can readily expect also that this relationship pattern will probably appear in the transference. Therapeutic abstinence in the sense of being silent and non-responding may instill more tension and anxiety. Technical neutrality in a modified way (e.g., Kernberg et al., 2008) and a warm, accepting and in general empathetic position at the right moment seems to be the most useful technique recommendation until the danger of a retraumatization is attenuated. According to Kernberg et al. (2008) technical neutrality might be an ideal point of departure within the treatment of traumatized patients, like Borderline patients, at large and within each session because it counters patients’ tendency to externalize their intrapsychic conflicts. However, at times it needs to be disrupted because of the urgent requirement for limit-setting and even in connection with the introduction of a major life problem of the patient that, at such point, would seem a non-neutral intervention of the therapist. Such deviation from technical neutrality may be indispensable in order to protect the boundaries of the treatment situation, and the patient from severe suicidal and other self-destructive behavior, and requires a particular approach in order to restore technical neutrality once it has been abandoned.

## CONCLUDING REMARKS

Our goal in this paper was to demonstrate the complexity of trauma-related disorders as informed by attachment theory and psychodynamic perspectives for the purpose of psychotherapy. The response to trauma is embedded in patients’ interpersonal difficulties and representations of self and attachment figures. Attachment representations should receive at least as much attention as their traumatic memories and symptoms (i.e., dissociative experiences dissociative defenses). The knowledge of the mental processes linked to traumatic dysregulation and disorganization of attachment should guide the therapist’s understanding of these difficulties. Some patients are frozen in a state of chronic pathological mourning, such as the case described here. Responses in other patients may take other forms, including other forms of mourning, such as preoccupation with personal suffering or failed mourning (Bowlby, 1980; George and West, 2012). The case analysis of Gloria, integrating the AAP with psychodynamic interpretation, demonstrates the phenomenological overlap and the developmental continuity between childhood attachment behavior and the behavior of adult dissociative patients within the therapeutic relationship (Liotti, 1993, 1995; Fonagy, 1999; Muscetta et al., 1999; Liotti and Intreccialagli, 2003). Correction of patients’ representations of attachment should become an important aim of the treatment.

One of the advantages of attachment assessment using the AAP is that the pictures scenes serve as stimuli for individual narratives. This means frightening memories can show up in a story without necessarily being articulated as one’s own experiences. Gloria’s predominant fear at the beginning of treatment was having to describe her trauma. She was so terrified that she abandoned therapy for quite a long time. Using the AAP in the context of an initial clinical assessment, the clinician gains specific knowledge about what kind of words might be eerie and traumatic for the patient due to his or her individual story. The clinician can then be more aware and careful in pursuing the details associated with a patient’s fear, which certainly will be reactivated in the therapeutic dyad. With the help of an understanding of individual traumatic dysregulation and defensive structure as provided by the AAP, therapeutic interventions can focus step by step on helping patients to understand their intense emotional reactions of helplessness in the context of the treatment setting. According to Fonagy and Bateman (2006), the patient must be helped to consider who engendered the feeling and how, and to explore whether the feelings have occurred or are connected to events either in the immediate or longer term past.

Gloria presented herself as a very strong and autonomous person. There was no evidence of this person in the AAP, which demonstrated that she did not truly view herself as autonomous. Gloria’s outward “strength” was controlling others, a strategy developed to manage frightening feelings of helplessness, abandonment, and isolation in the context of maltreatment and rejection (Solomon and George, 2011). Her strength was constructed from a disorganizing dissociation triangle and she knew and played out the script for each of its roles.

In thinking about the benefits of using the AAP in clinical settings, we must also discuss cautions and limitations. Developmental attachment assessments as designed to be stressful in order to be able to capture patterns of attachment representation and defensive processes. Using a picture set for assessment is a benefit of the AAP methodology, but the picture stimuli may become triggers, especially for individuals with PTSD. Because PTSD patients may be in a state of severe traumatization, it is possible that they may not be able to encode and respond to the task, which is called “constriction” (e.g., the person says that he or she is unable to create a story or hands the picture back to the interviewer saying they cannot or do not want to tell a story). Taking that into account, we stress that it would be very important to complete the AAP assessment in a supportive and caring clinical environment. Although more research is needed on the use of the AAP with disturbed patients, it is a promising instrument that has the potential to formulate psychodynamic hypotheses and treatment goals (see also Finn, 2011).

## Conflict of Interest Statement

The authors declare that the research was conducted in the absence of any commercial or financial relationships that could be construed as a potential conflict of interest.
